# PyMT-1099, a versatile murine cell model for EMT in breast cancer

**DOI:** 10.1038/s41598-018-30640-1

**Published:** 2018-08-14

**Authors:** Meera Saxena, Ravi Kiran Reddy Kalathur, Melanie Neutzner, Gerhard Christofori

**Affiliations:** Department of Biomedicine, University of Basel, Mattenstrasse 28, 4058 Basel, Switzerland

## Abstract

An epithelial-mesenchymal transition (EMT) has been implicated in cancer metastasis, drug resistance, and in conferring stem cell-like traits to cancer cells. Most studies investigating EMT in cancer have either utilized immortalized or cancer cell lines that are already primed to undergo an EMT and do not adequately represent a fully differentiated epithelial state in the absence of an EMT induction. Hence, model systems are required which recapitulate all stages of EMT in cancer cells. Here, we report the derivation and characterization of epithelial PyMT-1099 cancer cells from the MMTV-PyMT mouse model of breast cancer. We demonstrate that PyMT-1099 cells undergo an EMT upon TGFβ treatment, while upon TGFβ withdrawal they go through a mesenchymal-epithelial transition (MET), as assessed by changes in cell morphology and marker expression and comparable to normal murine mammary gland NMuMG cells. However, in contrast to NMuMG cells, PyMT-1099 cells show an increase in cell migration and are highly tumorigenic and metastatic when transplanted into immunocompromised mice. Finally, we report cancer cell-specific changes in gene expression during EMT of PyMT-1099 cells not found in non-transformed NMuMG cells. Thus, PyMT-1099 cells are a versatile tool to study breast cancer-associated EMT and MET *in vitro* and *in vivo*.

## Introduction

More than 90% of breast cancer-related deaths can be ascribed to systemic dissemination of cancer cells leading to their metastatic outgrowth in distant organs^1^. Since metastatic breast cancers are highly aggressive and drug resistant, understanding the molecular pathways underlying their malignant progression is critical for the development of efficacious cancer therapies. The evolutionarily conserved developmental program of an epithelial-mesenchymal transition (EMT), wherein cells lose their epithelial characteristics and instead acquire mesenchymal markers and cell motility, is believed to play an important role in the ‘initiation’ of the invasion-metastasis cascade of cancer cells^2–4^. An EMT is a physiological process during the multiple stages of embryogenesis and organ development, in which epithelial cells convert into highly mobile mesenchymal cells which give rise to bone, muscle, connective tissue, and blood vessels^5,6^. In malignant carcinogenesis, it is believed that cancer cells can hijack EMT to leave the primary tumor and invade into surrounding tissue and into blood and lymphatic vessels, providing a simple explanation for the seemingly complex process of cancer cell dissemination and metastasis formation^7,8^. Some recent reports have raised the possibility that specific EMT factors are responsible for specific stages of an EMT and for metastasis and drug resistance, thus suggesting the existence of specific subprograms and stages of partial and/or full EMT^7,9–13^. An EMT has also been implicated to overcome apoptosis induced by anchorage deprivation^14^ or to genotoxic stresses^15^. Finally, an EMT has been found to enrich for cancer stem-like cells (CSCs) that display mesenchymal characteristics and are resistant to chemotherapy^16,17^. However, to overcome the differences between experimental models and uncertainties about the various stages and the extent of an EMT and its contribution to cellular processes, there is a need of additional reliable model systems that can replicate the dynamic “plastic” changes associated with an EMT of cancer cells *in vitro* and *in vivo*.

Several cell lines are currently being used to study the process of an EMT *in vitro* and *in vivo*. Few examples include the normal murine mammary gland (NMuMG) cells^18^, Py2T cells^19^, the 4T1 cell series^20^, Madin-Darby canine kidney cells (MDCK) cells^21^ or the human cell lines MCF10A^22,23^, MCF7^24^ or immortalized human mammary epithelial (HMLE) cells^16^. While these cell lines are used across laboratories studying EMT, they are associated with some caveats. For example, while NMuMG, MDCK and MCF10A cells undergo an efficient transition from an epithelial to a mesenchymal phenotype in response to TGFβ *in vitro*, these cells are only immortalized and cannot be used to study a cancer-associated EMT *in vivo*. Py2T cells overcome this problem as they have been derived from a mammary gland tumor of a MMTV-PyMT transgenic mouse and can be transplanted into mice to study cancer cell EMT *in vivo*. However, Py2T cells exhibit a metastable epithelial phenotype and seem to have already initiated the EMT process in their basal state as evident by their morphology and EMT marker expression profile^19^. The widely used murine 67NR, 168FARN, 4TO7 and 4T1 cell series are useful for studying each step of the invasion-metastasis cascade individually^20^. However, none of these cell lines serve as an adequate model for a full EMT. Several studies have employed the highly aggressive human MDA-MB-231 cells to study EMT and its role in metastases formation *in vivo*^25^, but these cells are already in a stable mesenchymal state and lack the cell plasticity associated with an EMT. Hence, cellular models that recapitulate each dynamic step of an EMT and can also be used for tumor and metastasis formation experiments *in vivo* are rare.

We here report the generation and characterization of PyMT-1099, a murine cell line derived from a mammary tumor of a mouse mammary tumor virus- polyomavirus middle T Antigen (MMTV-PyMT) transgenic mouse^26^. PyMT-1099 cells undergo a TGFβ-induced EMT comparable to that of NMuMG (E9) cells *in vitro*, yet they form tumors and metastases upon transplantation into immunocompromised mice. In addition, PyMT-1099 cells undergo a complete mesenchymal to epithelial transition (MET) upon TGFβ withdrawal, similar to NMuMG (E9) cells. Hence, PyMT-1099 cells are an excellent model for EMT and MET research both *in vitro* and *in vivo*.

## Results

### Derivation of the PyMT-1099 cell line

**Figure 1:**
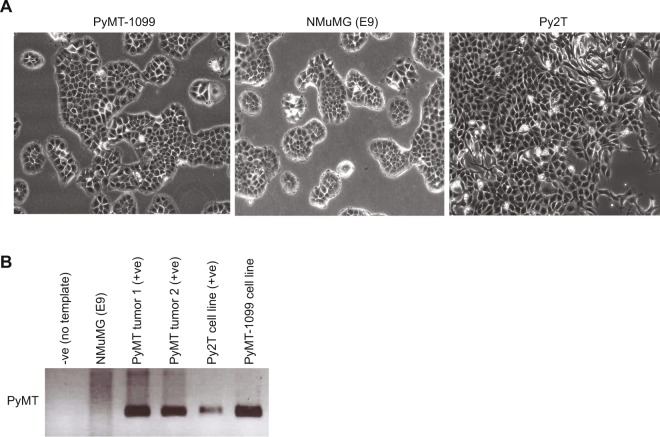
Generation of epithelial PyMT-1099 murine breast cancer cells. (**A**) Photomicrographs represent the morphology of epithelial PyMT-1099, NMuMG (E9) and Py2T cells. Magnification, 10X. (**B**) Genotyping PCR was performed on genomic DNA isolated from PyMT-1099 cells for the presence PyMT oncogene. NMuMG (E9) served as the negative control in addition to a no template control and PyMT primary tumors and Py2T cells served as the positive controls. Uncropped scans from the genotyping PCR (agarose gel) are displayed in Fig. S4.

To derive a cancer-specific cell line that could be used as an adequate model to study the process of an EMT *in vitro* and *in vivo*, we isolated cells from a mammary gland tumor of the MMTV-PyMT (FVB/N) transgenic mouse model of breast cancer^26^. Following mechanical and enzymatic dissociation, the cell mixture was plated in a 10 cm culture dish. After 1.5–2 months in culture with regular differential trypsinization to remove contaminating fibroblasts, individual clones started to proliferate and expand. Interestingly, these cells exhibited a well-differentiated, cobble stone-like morphology, very similar to the epithelial morphology of NMuMG (E9) cells. We named these cells PyMT-1099 (PyMT to refer to the mouse model and the number of the mouse from which the cells were derived) (Fig. 1A). In contrast, Py2T cells, though epithelial, lacked a defined cobble stone-like morphology and seemed to be in a “partial EMT” phenotype already (Fig. 1A), as previously reported^19^. The presence of the PyMT transgene in the genome of PyMT-1099 cells confirmed their tumor origin (Fig. 1B). The presence of the PyMT oncogene in two MMTV-PyMT primary breast tumors and Py2T cells served as positive controls, while its absence in NMuMG (E9) cells served as a negative control (Fig. 1B).

### TGFβ-induced EMT of PyMT-1099 cells *in vitro*

**Figure 2:**
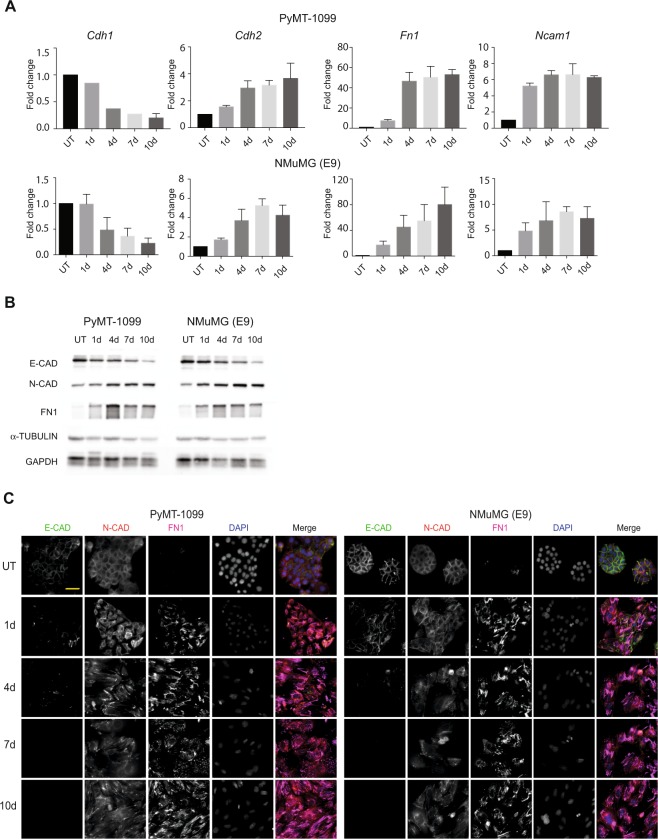
TGFβ-induced EMT in PyMT-1099 and NMuMG cells. PyMT-1099 (top of panel) and NMuMG (E9) (bottom of panel) cells were in parallel treated with TGFβ for 0 (UT), 1, 4, 7 or 10 days. (**A**) RNA isolated from the cells was subjected to quantitative RT-PCR analyses of EMT markers. Graphs represent the relative RNA expression levels of epithelial marker, *Cdh1* and mesenchymal markers *Cdh2*, *Fn1* and *Ncam1* normalized the housekeeping gene *Rpl19*; n = 3. (**B**) Immunoblotting analyses was performed to assess the protein expression levels of epithelial marker E-CAD and mesenchymal markers N-CAD and FN1. α-TUBULIN was used as the loading control; n = 3. All samples were run in parallel on the same gel. Uncropped immunoblot scans from main blots are displayed in Fig. S5. (**C**) Immunofluorescence analysis was performed to assess the expression and/or localization of EMT markers E-CAD, N-CAD and FN1; n = 3. DAPI was used as a nuclear counterstain. Scale bar, 50 μm.

To assess whether PyMT-1099 cells could undergo the morphogenic events associated with an EMT, they were cultured in the presence of TGFβ for 1, 4, 7 or 10 days. Interestingly, comparable to immortalized NMuMG (E9) cells, PyMT-1099 cells exhibited a clear change from epithelial to mesenchymal morphology during 10 days of TGFβ treatment (see Supplementary Fig. S1A). Quantitative RT-PCR analysis revealed a decrease in the expression of epithelial marker E-cadherin (*Cdh1*), and an increase in the expression of mesenchymal markers, such as N-cadherin (*Cdh2*), fibronectin 1 (*Fn1*) and neural cell adhesion molecule (*Ncam1*) in PyMT-1099 cells treated during 10 days with TGFβ (Fig. 2A). Comparable quantitative changes in the expression of epithelial and mesenchymal markers were also observed in NMuMG (E9) cells upon TGFβ treatment (Fig. 2A).

The results above were further confirmed by immunoblotting for the epithelial marker E-cadherin (E-CAD) and the mesenchymal markers N-cadherin (N-CAD) and FN1 (Fig. 2B). Finally, immunofluorescence microscopy analysis revealed that the epithelial markers E-cadherin (E-CAD) and zona occludens 1 (ZO-1) completely vanished from the cell membranes (Fig. 2C, Supplementary Fig. S1B), while the mesenchymal marker FN1 was significantly and N-CAD moderately upregulated in both PyMT-1099 and NMuMG (E9) cells over the 10 days of TGFβ treatment (Fig. 2C). The formation of focal adhesions (assessed by Paxillin1 immunofluorescence) and rearrangement of cortical actin into stress fibers (assessed by phalloidin immunofluorescence), additional hallmarks of an EMT, confirmed that PyMT-1099 cells undergo a TGFβ-induced EMT highly comparable to NMuMG (E9) cells (see Supplementary Fig. S1B). In addition, removal of TGFβ leads to a mesenchymal-epithelial transition (MET) over 7–10 days, comparable to NMuMG (E9) cells, as assessed by cell morphology (see Supplementary Fig. S1C). Collectively, these results demonstrate that PyMT-1099 murine breast cancer cells are a comparably valid model to study TGFβ-induced EMT and MET as the frequently used non-transformed NMuMG cells.

### PyMT-1099 cells undergo a functional EMT *in vitro*

**Figure 3:**
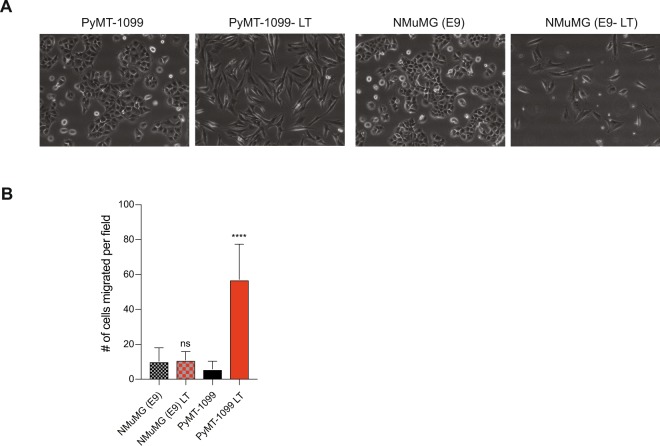
Long-term TGFβ treatment and cell migration of PyMT-1099 and NMuMG cells. PyMT-1099 and NMuMG (E9) cells were treated for >20 days with TGFβ to create long term (LT) mesenchymal cells. (**A**) Photomicrographs represent the morphology of untreated parental and LT PyMT-1099 and NMuMG (E9) cells. Magnification, 10X. (**B**) Graph represents the number of cells migrated per field across the membrane in a Boyden chamber transwell migration assay; n = 3. ****signifies p-value < 0.0001.

A functional EMT can be defined as the ability of cells that have undergone an EMT to efficiently migrate and invade in appropriate *in vitro* assays or by their ability to locally invade, enter the blood circulation and form distant metastases at secondary sites *in vivo*. To assess their migratory potential, untreated and >20 days TGFβ-treated PyMT-1099 cells (PyMT-1099 LT) were subjected to a modified Boyden chamber transwell migration assay. Untreated and >20 days TGFβ-treated NMuMG cells (NMuMG (E9) LT) were in parallel subjected to the migration assay as well. Long-term TGFβ treatment caused both PyMT-1099 and NMuMG (E9) cells to attain a mesenchymal cell morphology, as compared to the epithelial morphology of the untreated cells (Fig. 3A). While TGFβ-treated, mesenchymal PyMT-1099 cells had a significantly higher migratory potential compared to their epithelial counterparts, TGFβ treatment of NMuMG (E9) cells failed to induce a significant increase in their migration (Fig. 3B). These results reveal PyMT-1099 cells as an adequate model to assess a functional EMT of mammary cancer cells *in vitro*, while non-transformed NMuMG cells lack this quality.

### PyMT-1099 cells are highly tumorigenic and metastatic

**Figure 4:**
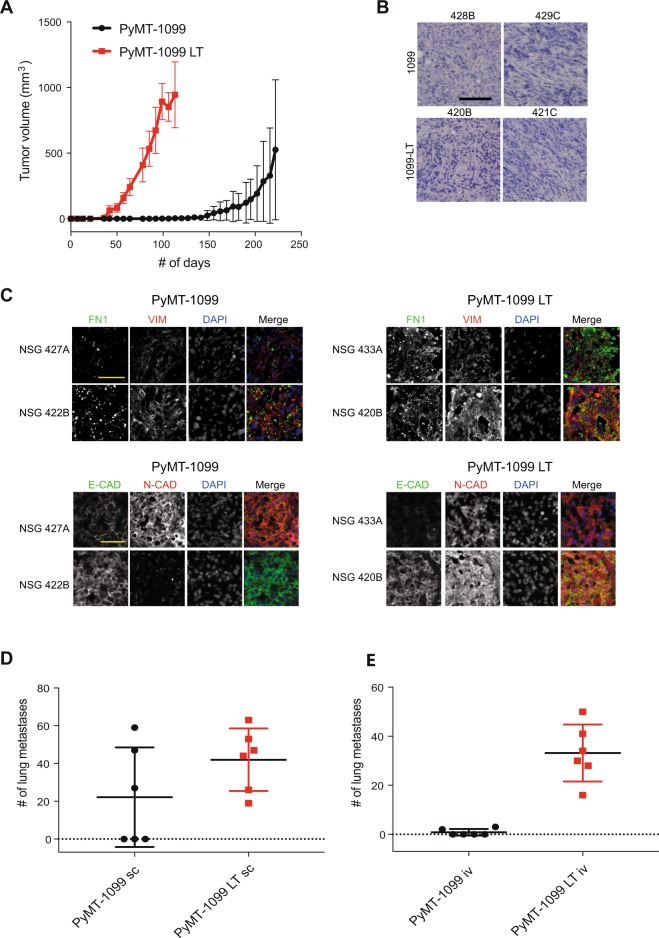
Tumor formation and metastasis by PyMT-1099 cells. (**A**) PyMT-1099 cells untreated or treated with TGFβ for >20 days (PyMT-1099 LT) were injected orthotopically into mammary fat pads of NSG mice. The graph represents tumor growth in PyMT-1099 and PyMT-1099 LT group of mice. **(B)** Histological tumor sections from tumors of PyMT-1099 or PyMT-1099 LT cells described in (**A**) were stained with H&E to assess the morphology of primary tumors. Representative microphotographs are shown from tumors of 2 out of the 6 mice used in the experiment. (**C**) Immunofluorescence analysis was performed to assess the expression of the EMT markers FN1, VIM, E-CAD and N-CAD in tumors formed by PyMT-1099 or PyMT-1099 LT cells in the experiment described in (**A**). DAPI was used as a nuclear counterstain. Representative pictures are shown from tumors of 2 out of the 6 mice used in the experiment. Scale bar, 100 μm. (**D**) The graph represents the number of lung metastases formed in NSG mice orthotopically transplanted with PyMT-1099 or PyMT-1099 LT cells; n = 6. (**E**) The graph represents the number of lung metastases formed in NSG mice injected with PyMT-1099 or PyMT-1099 LT cells through the tail vein; n = 6. The mice were sacrificed 8 weeks post-injection, and lungs were resected for the analysis of cancer cell colonization/ metastases formation.

To assess the tumorigenicity of PyMT-1099 cells, untreated and long-term TGFβ-treated (LT) PyMT-1099 cells were orthotopically implanted into the mammary fat pad of immunodeficient NOD/SCID; common gamma receptor −/− (NSG) mice. Mesenchymal PyMT-1099 LT cells formed palpable tumors at 5 weeks (~36 days) of injection and these tumors grew rapidly until 16 weeks (~113days), when mice had to be sacrificed due to ethical termination criteria (Fig. 4A). Macroscopic metastases were visible in the lungs of all of these mice (data not shown). In comparison, tumors of non-treated, epithelial PyMT-1099 cells formed palpable tumors only after 14 weeks (~99 days) post-injection (Fig. 4A). These mice had to be sacrificed around 32 weeks (~222 days) post-injection, and macroscopic metastases were only found in three out of six mice (data not shown). Untreated or TGFβ-treated PyMT-1099 cells did not give rise to tumors upon orthotopic injection in syngenic FVB/N mice (data not shown), most likely due to an immunological rejection of the PyMT transgene. Histopathological analyses of the primary tumors formed by PyMT-1099 LT cells revealed the presence of mesenchymal cells with elongated nuclei invading through the surrounding stroma (Fig. 4B, Supplementary Fig. S2A). Three tumors formed by non-treated PyMT-1099 cells also contained scattered mesenchymal cells invading through the stroma (mice numbers 429 C, 442D and 444 F), while the other three tumors contained mainly highly differentiated epithelial cells (mouse numbers 428B, 427 A and 443E) (Fig. 4B, Supplementary Fig. S2A).

Next, we went on to characterize the expression of EMT-related markers in the primary tumors formed by parental PyMT-1099 or by long-term TGFβ-treated PyMT-1099 LT cells. As expected, all the six mice in the PyMT-1099 LT group had formed tumors that expressed the mesenchymal markers FN1, Vimentin (VIM), and N-CAD at high levels (Fig. 4C, Supplementary Fig. S2B). E-CAD expression could also be detected at various levels in these tumors (Fig. 4C, Supplementary Fig. S2B). The tumors formed by the mice in the parental PyMT-1099 group showed a more heterogenous expression of EMT-related markers. They also expressed the mesenchymal markers FN1, VIM and N-CAD albeit to a lower level compared to PyMT-1099 LT tumors (Fig. 4C, Supplementary Fig. S2B). Like PyMT-1099 LT tumors, PyMT-1099 tumors also expressed E-CAD heterogeneously to different extents (Fig. 4C, Supplementary Fig. S2B).

We next assessed the number of lung metastases formed in the mice from the experiment above, where cells had been orthotopically injected into the mammary fat pad of NSG mice. Interestingly, histopathological analysis revealed that all the six mice injected with PyMT-1099 LT cells carried lungs full of metastases (Fig. 4D). In the PyMT-1099 group, however, only three out of six mice exhibited lung metastases (Fig. 4D). Interestingly, the mice of the PyMT-1099 group with metastases were the ones which showed invasive primary tumors (Fig. 4B, Supplementary Fig. S2A). Next, in a lung colonization assay, we injected PyMT-1099 parental and PyMT-1099 LT cells into the tail vein of mice and assessed the formation of lung metastases after 8 weeks. As expected, PyMT-1099 LT cells were able to efficiently colonize the lungs of all 6 mice in the group, while only 2 mice in the PyMT-1099 group yielded 2–3 metastases (Fig. 4E). In contrast, due to immunological rejection, lung colonization of PyMT-1099 parental and LT cells in syngeneic FVB/N mice was inefficient. Only 3 out of 6 mice from the LT group yielded 2–3 metastases and only 1 out of 6 mice in the parental group yielded just one metastasis (see Supplementary Fig. S2C). These results collectively suggest that PyMT-1099 cells are a suitable model to study primary tumor growth and invasion, tumor cell EMT, and the formation of lung metastasis in immunocompromised mice *in vivo*. Metastasis in other distant organs was not detected in the mice examined.

### Cancer-specific changes in gene expression during an EMT

**Figure 5:**
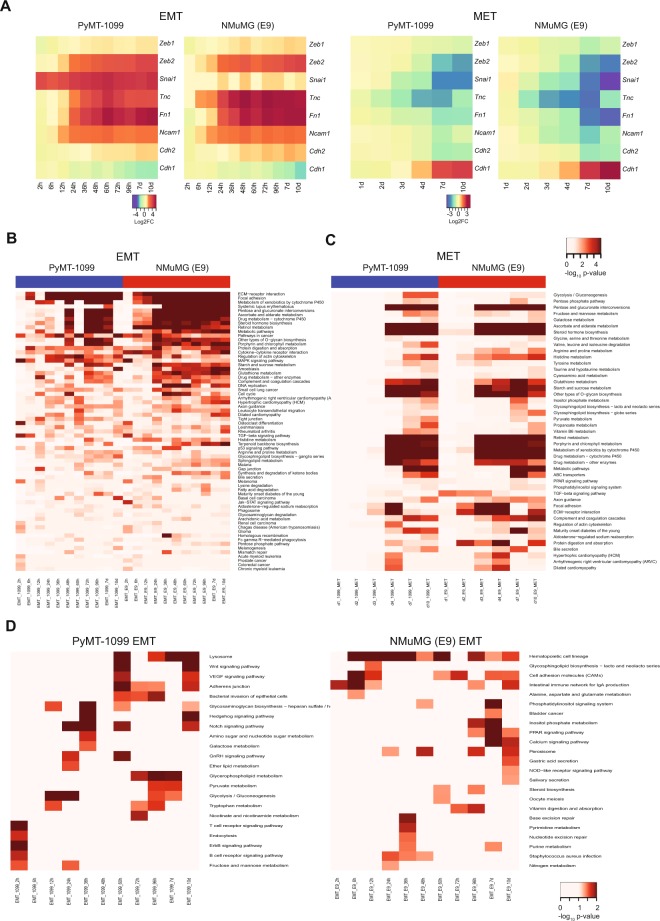
Gene expression analysis of a TGFβ-induced EMT in PyMT-1099 and NMuMG cells. (**A**) The heatmaps represent expression of selected EMT markers during the EMT and MET time courses in PyMT-1099 and NMuMG (E9) cells as determined by RNA-Seq analysis. (**B**) The heatmap represents the “common” significantly regulated pathways during TGFβ−induced EMT time courses in PyMT-1099 and NMuMG (E9) cells obtained by GO analysis of the RNA-Seq data. (**C**) The heatmap represents the “common” significantly regulated pathways during MET time courses in PyMT-1099 and NMuMG (E9) cells obtained by GO analysis of the RNA-Seq data. (**D)** The heatmaps represent the “unique” significantly regulated pathways during the TGFβ-induced EMT time courses in PyMT-1099 and NMuMG (E9) cells computed by GO analysis of the RNA-Seq data.

To assess the transcriptional changes occurring during an EMT, PyMT-1099 and NMuMG (E9) cells were treated with TGFβ in a detailed EMT time-course followed by RNA sequencing (RNA-Seq). TGFβ was also withdrawn from 10 days pretreated cells over a time-course (see Supplementary Fig. S1C) to determine the transcriptional changes underlying an MET. Correlation (see Supplementary Fig. S3A) and PCA analysis (see Supplementary Fig. S3B) revealed high quality and reproducibility between the two biological replicates of all samples. Volcano plots depict the number of differentially regulated genes in the EMT and MET time courses of PyMT-1099 cells (see Supplementary Fig. S3C, D). Analysis of the expression of selected EMT-related genes, such as Zinc Finger E-Box Binding Homeobox 1 (*Zeb1)*, Zinc Finger E-Box Binding Homeobox 2 (*Zeb2)*, Snail1 (*Snai1)*, Tenascin C (*Tnc)*, fibronectin (*Fn1)*, neural cell adhesion molecule 1 (*Ncam1)*, E-cadherin (*Cdh1)* and N-cadherin (*Cdh2)* confirmed that PyMT-1099 and NMuMG (E9) cells undergo comparable changes in gene expression during EMT and MET (Fig. 5A). Further, functional enrichment analysis on differentially expressed genes revealed pathways significantly enriched during an EMT in both PyMT-1099 and NMuMG (E9) cells, including ECM-receptor interactions, focal adhesions, TGFβ signaling and regulation of actin cytoskeleton and many more (Fig. 5B). Likewise, during a MET of both cell types, many similar pathways were found to be significantly enriched, in particular, massive metabolic reprogramming (Fig. 5C). Importantly, unique pathways were identified that were significantly regulated during an EMT in PyMT-1099 breast cancer cells and not in non-transformed NMuMG (E9) cells, for instance vascular endothelial growth factor (VEGF) signaling, Wnt signaling and Notch signaling (Fig. 5D). These pathways may make a difference between an EMT of normal/immortalized cells as compared to an EMT of cancer cells, yet further in-depth data analysis and experimental validation are required to test this hypothesis.

## Discussion

An epithelial-mesenchymal transition (EMT) has been suggested to play various roles in malignant carcinogenesis, from initiating the invasion-metastasis cascade and promoting metastasis formation^6^ to functional contributions in cell survival, drug resistance and stem cell-like properties of cancer cells^7,10,13^. To deepen our understanding of an EMT in cancer, it is important to have at hand an experimental system that mimics each stage of an EMT *in vitro* and *in vivo*. We report the derivation of PyMT-1099, an epithelial cancer cell line from a murine model of breast cancer that recapitulates the morphogenetic changes associated with the stepwise progression of an EMT and also MET, enabling to study not only the extreme epithelial or mesenchymal states, but also the plasticity of the transitioning states associated with a partial EMT/MET. Along with the transcriptomic profiling by RNA-Seq of high resolution EMT and MET time-courses, we also provide the research community with data available to study one’s gene of interest in the context of a dynamic EMT/MET.

We show that PyMT-1099 cells, highly similar to NMuMG (E9) cells, show a fully differentiated epithelial phenotype and undergo an EMT upon treatment with TGFβ, as assessed by cell morphology and by a variety of EMT marker expression. Further, similar to NMuMG (E9) cells, PyMT-1099 cells undergo a complete MET upon removal of TGFβ over a time-course of 7–10 days (see Supplementary Fig. S1C) and hence can also serve as an excellent model to study processes regulating the reversibility of EMT. In addition, PyMT-1099 cells, unlike NMuMG (E9) cells, can also undergo a functional EMT as they attain motility and migrate in response to TGFβ treatment *in vitro*. Further, since PyMT-1099 cells are transformed cancer cells, they are also able to form tumors and metastases upon transplantation into immunodeficient NSG mice *in vivo*. Thus, while NMuMG (E9) cells remain an excellent model for the dynamic process of an EMT, they stay within the limitation of a non-tumorigenic cell system. Other models being currently used to study EMT in cancer *in vivo*, such as murine 4T1 or human MDA-MB-231 breast cancer cells, suffer from the disadvantage of being partially or completely mesenchymal at their baseline and, hence, they do not yield accurate information on the full dynamics of EMT-related processes. Py2T cells in this regard are better than the aforementioned cells, however, they are also already in the first phases of an EMT-like program by already co-expressing epithelial and mesenchymal markers. PyMT-1099 cells overcome this drawback by exhibiting a complete epithelial phenotype under normal culture conditions.

Tumors formed by PyMT-1099 cells implanted into the mammary fat pads of NSG mice express the mesenchymal markers FN1, VIM and N-CAD and epithelial marker E-CAD (Fig. 4C, Supplementary Fig. S2B). Hence, even though completely epithelial cells were injected into the mice, these cells undergo a partial EMT as suggested by the co-expression of epithelial and mesenchymal markers. When scored for formation of lung metastases, only three out of six mice developed metastatic lesions in the lung. Interestingly the mice that carried metastases were the ones with invasive primary tumors (Fig. 4B, Supplementary Fig. S2A), suggesting that the invasive phenotype in the primary tumors correlates with the ability of cancer cells to form metastases at secondary sites.

Tumors formed by PyMT-1099 cells treated with TGFβ for >20 days (PyMT-1099 LT) express mesenchymal markers FN1, VIM and N-CAD at high levels (Fig. 4C, Supplementary Fig. S2B). Even though at the time of injection these cells were completely mesenchymal, tumors formed by the LT cells re-express E-CAD at varying levels (Fig. 4C, Supplementary Fig. S2B), suggesting that the mesenchymal cancer cells need to re-express epithelial markers for clonal outgrowth. All of the 6 mice in the PyMT-1099 LT group developed large numbers of lung metastases. Both PyMT-1099 and PyMT-1099 LT cells fail to efficiently form tumors or metastases in syngeneic FVB/N mice, the genetic background from which they have been originally derived. This is likely due to their rejection by the expression of the PyMT oncogene, as has been observed for comparable cell lines before^27^.

Transcriptomic profiling by RNA sequencing of a detailed time course of TGFβ-induced EMT as well as TGFβ removal-induced MET reveals a number of molecular pathways and cellular processes shared between PyMT-1099 cells and NMuMG cells, such as ECM-receptor interactions, focal adhesion, TGFβ signaling and actin cytoskeleton remodeling. Hence, with regard to general processes underlying an EMT or MET, transformed PyMT-1099 breast cancer cells and non-transformed NMuMG mammary gland epithelial cells seem to be highly comparable. However, several pathways differ between them, including signaling by VEGF^28^, Wnt^29,30^ and Notch^31^, pathways which are frequently dysregulated in cancer. The novel observation of commonalities but also differences between EMT in cancer cells as compared to their non-transformed counterparts provokes further investigations into their functional contributions to the differences in EMT/MET between a normal physiological process versus malignant cancer progression.

## Conclusions

We report the generation of a murine, epithelial breast cancer cell line, PyMT-1099, that serves as a versatile experimental system to model all stages across the EMT and MET spectrum and enables investigations into how an EMT or a MET are orchestrated and also how they contribute to cancer cell invasion and metastasis, drug resistance, stress survival and stemness both *in vitro* and *in vivo*.

## Materials and Methods

### Antibodies and Reagents

E-cadherin (BD Transduction Labs, 610182; used for immunoblotting), E-cadherin (Zymed, 13-1900; used for immunofluorescence stainings), N-cadherin (Takara, M142), Zona Occludens-1 (Zymed, 617300), Paxillin (BD, 610052), Fibronectin1 (Sigma-Aldrich, F3648), Vimentin (Novus Biological, NB300–223), α-Tubulin (Sigma, T-9026), GAPDH (Abcam, ab9485), Alexa-Fluor 488 and 568 (Molecular Probes), secondary horse radish peroxidase (HRP)-conjugated antibodies against mouse and rabbit (Jackson ImmunoResearch), Phalloidin Alexa-Fluor 568 (Molecular Probes, A12380), 4′,6-diamidino-2-phenylindole (DAPI, Sigma-Aldrich, D9542), recombinant human TGFβ1 (R&D Systems, 240-B).

### Cell culture

Murine breast epithelial cell lines NMuMG (E9- epithelial clone 9)^18^, Py2T^19^ and PyMT-1099 were cultured in Dulbecco’s modified Eagle’s medium (DMEM) (Sigma-Aldrich, D5671) supplemented with 10% Fetal Bovine Serum (Sigma-Aldrich, F7524), 2 mM glutamine (Sigma-Aldrich, G7513), 100 U penicillin (Sigma-Aldrich) and 0.1 mg/ml streptomycin (Sigma-Aldrich). All cell lines were grown at 37 °C, 5% CO_2_, 95% humidity. For EMT experiments, cells were treated with 2 ng/ml TGFβ1 for the time points indicated.

### PyMT-1099 cell line derivation

PyMT-1099 cells were isolated from a mammary gland tumor (mammary gland 2/3) of a MMTV-PyMT transgenic female mouse (FVB/N background)^26^. In brief, a small piece of a tumor was minced and subjected to enzymatic digestion with 0.1 mg/ml DNaseI (Roche, 11284932001) and 1 mg/ml Collagenase D (Roche, 11088858001) supplemented with 50 μg/ml gentamycin (Sigma, G1397) and 1X antibiotic-antimycotic (ThermoFisher, 15240062) for 30 min in sterile conditions. The cell mixture was passed through a 70 μm cell strainer (BD Falcon, 352350) and the single cells obtained were plated as a polyclonal population in a 10 cm dish in DMEM supplemented with 10% FBS (Sigma), 10% horse serum (Amimed), 100 U penicillin (Sigma-Aldrich) and 0.1 mg/ml streptomycin (Sigma-Aldrich). Medium was changed regularly and any fibroblasts in culture were removed by several passages of differential trypsinization until only epithelial cells remained. PyMT-1099 cells were thereof cultured in DMEM supplemented with glutamine, penicillin, streptomycin, and 10% FBS (Sigma). Isolation of this cell line was done with approval, and according to the rules and guidelines of the Swiss Federal Veterinary Office (SFVO) and the local ethics committee (Cantonal Veterinary Office, Basel-Stadt, Switzerland; license 1878).

### Genotyping

To extract genomic DNA, cells grown on a confluent 10 cm petri dish were trypsinized, washed in PBS and pelleted. To the pellet, 250 μl of Buffer B (20 mM Tris-Cl pH 7.4, 4 mM EDTA, 10 mM NaCl) and 5 μl of 20 mg/ml Proteinase K were added. Following incubation at 55 °C for 2 h, 200 μl 5.3 M NaCl was added and samples were centrifuged at 13,000 g for 20 min at 4 °C. The supernatant was transferred to a fresh tube and DNA was precipitated by adding equal volume of cold isopropanol. Samples were centrifuged at 13,000 g for 20 min at 4 °C and the pellet was washed with 70% ethanol. The DNA pellet was dried for 15 min at 37 °C and then suspended in 100 μl of TE buffer. The samples were analyzed using standard PCR procedures. The nucleotide sequences of the primers used for genotyping the PyMT transgene are listed in Supplementary Table S1. Uncropped scans from the genotyping PCR (agarose gel) are displayed in Supplementary Fig. S4.

### RNA isolation and real-time qPCR

Total RNA was isolated using the guanidine isothiocyanate and phenol/chloroform method from cells harvested with TRI reagent (Sigma-Aldrich). Reverse transcription of mRNA was carried out using ImProm-II™ Reverse Transcription System (Promega, A3803) according to the manufacturer’s instructions. mRNA levels were quantified by real-time qPCR using PowerUp SYBR Green Master Mix (ThermoFisher, A25743) according to the manufacturer’s instructions. Mouse Riboprotein L19 (mRPL19) primers were used for normalization. qPCR assays were performed in duplicates, and fold changes were calculated using the comparative Ct method (ΔΔCt). Sequences of the specific primers used in the study are listed in Supplementary Table S1.

### Immunoblotting

Cells were lysed on ice for 30 min in RIPA buffer (50 mM Tris-HCl (pH8.0), 150 mM NaCl, 10% glycerol, 1% NP40, 0.5% sodium deoxycholate, 0.1% SDS, 2 mM MgCl_2_, 2 mM CaCl_2_) with 1 mM DTT, 1 mM NaF, 2 mM sodium orthovanadate and 1X protease inhibitor cocktail (Sigma-Aldrich) followed by scraping into tubes and centrifugation for 10 min at 10,000 rpm at 4 °C. Protein concentration in the supernatants was determined using Bio-Rad Bradford solution according to the manufacturer’s instructions. Proteins were mixed with 1X Laemmli sample buffer and equal amounts were size-fractionated on a SDS polyacrylamide gel. Proteins were then transferred onto an Immobilon-P PVDF membrane (Millipore) using the wet transfer method for 2 h at constant current (0.4 A). Following blocking for 1 h in 5% skimmed milk prepared in TBS/0.05% Tween 20, the membranes were incubated with appropriate primary antibodies overnight at 4 °C. Next day, after washes, the blots were incubated with HRP-conjugated secondary antibodies for 1 h at room temperature and visualized with Immobilon Western Chemiluminescent HRP Substrate (Millipore, WBKLS0500) on a Fusion Fx7 chemiluminescence reader. Uncropped immunoblot scans from main blots are displayed in Supplementary Fig. S5.

### Transwell migration assay

50,000 untreated or long term (>20d) TGFβ treated cells were suspended in 500 μl of DMEM/ 0.2% FBS and seeded into 24 trans-well migration inserts (Corning, 353097) in duplicates. The bottom chambers were filled with 700 μl of DMEM/ 20% FBS to create a chemo-attractant gradient. The cells were incubated in a tissue culture incubator at 37 °C with 5% CO_2_. After 18 h, inserts were fixed with 4% paraformaldehyde for 10 min. Cells that had not crossed the membrane were removed with a cotton swab, and cells on the bottom of the membrane were stained with DAPI. Images of five fields per insert were taken with a Leica DMI 4000 microscope and stained cells were counted using an ImageJ software plugin developed in-house.

### Immunofluorescence of cultured cells

Cells were grown on uncovered glass coverslips (#1, 12 mm round, Menzel-Glaser) and treated for the indicated times with TGFβ. Cells were fixed with 4% paraformaldehyde for 20 min at room temperature, followed by permeabilization with 0.5% NP40 for 5 min and blocking with 3% BSA/0.01% Triton X-100/PBS for 30 min. Cells were then incubated with primary antibodies diluted in blocking solution at room temperature for 2 h followed by incubation with a fluorophore-coupled secondary antibody (Alexa Fluor, Invitrogen) for 1 h at room temperature in dark. Cell nuclei were counterstained with DAPI (Sigma-Aldrich, D9542). After staining, the coverslips were mounted in fluorescence mounting medium (Dako, S302380-2) on microscope slides and imaged using a fluorescence microscope (Leica DMI 4000).

### Immunofluorescence of tissue sections

For immunohistochemistry, tumors were fixed at 4 °C in 4% paraformaldehyde for 2 h followed by cryopreservation overnight in 20% sucrose/PBS prior to embedding in OCT-Compound freezing matrix. For immunofluorescence analysis, cryosections were cut at 7 μm thickness and dried for 30 min prior to rehydration in PBS. Tissue sections were permeabilized with 0.2% TritonX-100/PBS and blocked for 30 min in 5% normal goat serum/PBS followed by incubation with the primary antibody in blocking buffer overnight at 4 °C. Next day sections were incubated with fluorophore-coupled secondary antibody (Alexa Fluor, Invitrogen) for 1 h at room temperature in dark. Cell nuclei were counterstained with DAPI (Sigma-Aldrich). After staining, the coverslips were mounted in fluorescence mounting medium (Dako, S302380-2) on microscope slides and imaged using a fluorescence microscope (Leica DMI 4000).

### Tissue histology

For histolopathological analysis, tissues (mammary tumors or lungs) were fixed in 4% paraformaldehyde overnight at 4 °C followed by embedding in paraffin after ethanol/xylene dehydration. Paraffin-embedded samples were cut at 5 μm thickness and subjected to Hematoxylin and Eosin (H&E) staining. To quantitate lung metastases, 9 slides per organ spaced 50 μm were stained with H&E and number of metastases in the lungs were counted under the microscope at 10 X magnification. Photomicrographs of mammary gland tumors were captured on a Zeiss Axio Imager Scanning Microscope at 10 X magnification with ZEN software.

### Tumor cell transplantation

For orthotopic implantations, 11–13 weeks old immunocompromised NOD/SCID;common gamma receptor −/− (NSG) female mice were anesthetized with isoflurane and injected with 1 × 10^6^ PyMT-1099 or PyMT-1099 long term (LT) cells in 100 µl Matrigel plus PBS (1:1) in the 9^th^ mammary fat pad. Tumors were measured during their progression and mice were sacrificed when either the tumors reached the total volume of 1.5 cm^3^ or when mice appeared unhealthy during the course of the experiment, whichever was earlier. 6 mice per group were used. Tumor volume was calculated according to the formula V = 0.543 × (L × W × W) where L represents length and W represents width of tumors measured by a digital Vernier caliper.

For tail vein injections, 6–8 weeks old NSG or FVB/N (female) mice were injected with 1 × 10^6^ tumorigenic cells in 100 µl PBS into the tail vein (intravenously). Mice were routinely observed for general health status. Mice were sacrificed 8 weeks post-injection and lungs were assessed for metastases formation histologically. 6 mice per group were used. All studies involving mice were approved by the Swiss Federal Veterinary Office (SFVO) and the regulations of the Cantonal Veterinary Office of Basel Stadt (license 1907). Animal experiments were performed in strict accordance with the guidelines of the Swiss Federal Veterinary Office (SFVO) and the regulations of the Cantonal Veterinary Office of Basel-Stadt (license number 1907). During the whole course of animal experiments, all efforts were made to minimize suffering.

### RNA sequencing analysis

For the EMT time-course, PyMT-1099 or NMuMG (E9) cells were treated with 2 ng/ml TGFβ for 2 h, 6 h, 12 h, 24 h, 36 h, 48 h, 72 h, 96 h, 7d or 10d. Untreated cells served as control. For the MET time course, TGFβ was withdrawn from PyMT-1099 or NMuMG (E9) cells after 10d of treatment. Cells were then seeded without TGFβ for 1, 2, 3, 4, 7 or 10d. 10d TGFβ treated cells (MET d0) served as the control for MET experiment. Biological duplicates were prepared for RNA sequencing.

Total RNA was isolated from the samples above using the miRNeasy Mini Kit (Qiagen, 217004) with on-column DNAse digestion according to the manufacturer’s instructions. RNA quality control was performed with an RNA ScreenTape on the Agilent 4200 TapeStation and the concentration was measured by using the Quanti-iT RiboGreen RNA assay Kit (Life Technologies). 200 ng of RNA was subjected to rRNA depletion and utilized for library preparation with the Truseq Stranded Total RNA Library Prep kit with Ribo-Zero Gold (Illumina). Library QC was performed with a Fragment Analyzer (AATI) using the Standard Sensitivity NGS Fragment Analysis Kit (DNF-473). RNA-Seq libraries were sequenced SR81 with NextSeq. 500 High Output v2 kit (Illumina) on an Illumina NextSeq 500 using protocols defined by the manufacturer. Primary data analysis was done using Illumina RTA Version 2.4.11.

Single-end RNA-seq reads (81-mers) were mapped to the mouse genome assembly, version mm10, with RNA-STAR^32^, with default parameters except for allowing only unique hits to genome (outFilterMultimapNmax = 1) and filtering reads without evidence in spliced junction table (outFilterType = “BySJout”). Expression levels per gene (counts over exons) for the RefSeq mRNA coordinates from UCSC (genome.ucsc.edu, downloaded in December 2015) were quantified using qCount function from QuasR package (version 1.12.0)^33^. The differentially expressed genes were identified using the edgeR package (version 3.14.0)^34^. Genes with p-value ≤ 0.05 and minimum log2 fold change of +/−0.58 were considered as differentially regulated and were used for downstream functional and pathway enrichment analysis.

### Functional enrichment analysis

Functional enrichment analysis of differentially expressed genes for biological processes or pathways were performed in R using several publicly available Bioconductor resources, including org.Ms.eg.db (version 3.6.0), GO.db (version 3.4.1), GOstats (version 2.42.0)^35^, KEGG.db (version 3.2.3) and ReactomePA (version 1.16.2)^36^. The significance of each biological processes or pathways identified was calculated using the hypergeometric test (equivalent to Fisher’s exact test) and those with p-values ≤ 0.05 were considered significant.

### Statistical analysis

Statistical analysis was performed using GraphPad Prism 7.0 software. All data are presented as mean ± S.E.M. p-values < 0.05 were considered statistically significant. All experiments were repeated thrice, unless otherwise stated.

## Electronic supplementary material

Supplementary Information

## Data Availability

The datasets generated and/or analyzed during the current study are deposited at Gene Expression Omnibus (GEO, accession numbers: GSE112797 (NMuMG (E9) EMT RNA-Seq data); GSE117474 (NMuMG (E9) MET RNA-Seq data); GSE1145722 (PyMT-1099 EMT and MET RNA-Seq data).
