# Author Correction: PyMT-1099, a versatile murine cell model for EMT in breast cancer

**DOI:** 10.1038/s41598-020-68523-z

**Published:** 2020-07-07

**Authors:** Meera Saxena, Ravi Kiran Reddy Kalathur, Melanie Neutzner, Gerhard Christofori

**Affiliations:** Department of Biomedicine, University of Basel, Mattenstrasse 28, 4058 Basel, Switzerland

Correction to:* Scientific Reports* 10.1038/s41598-018-30640-1, published online 14 August 2018

In Figure 2C, the merge image of immunofluorescent staining of NMuMG (E9) at 7 days is incorrect. The correct Figure 2 appears below as Figure 1.

**Figure 1:**
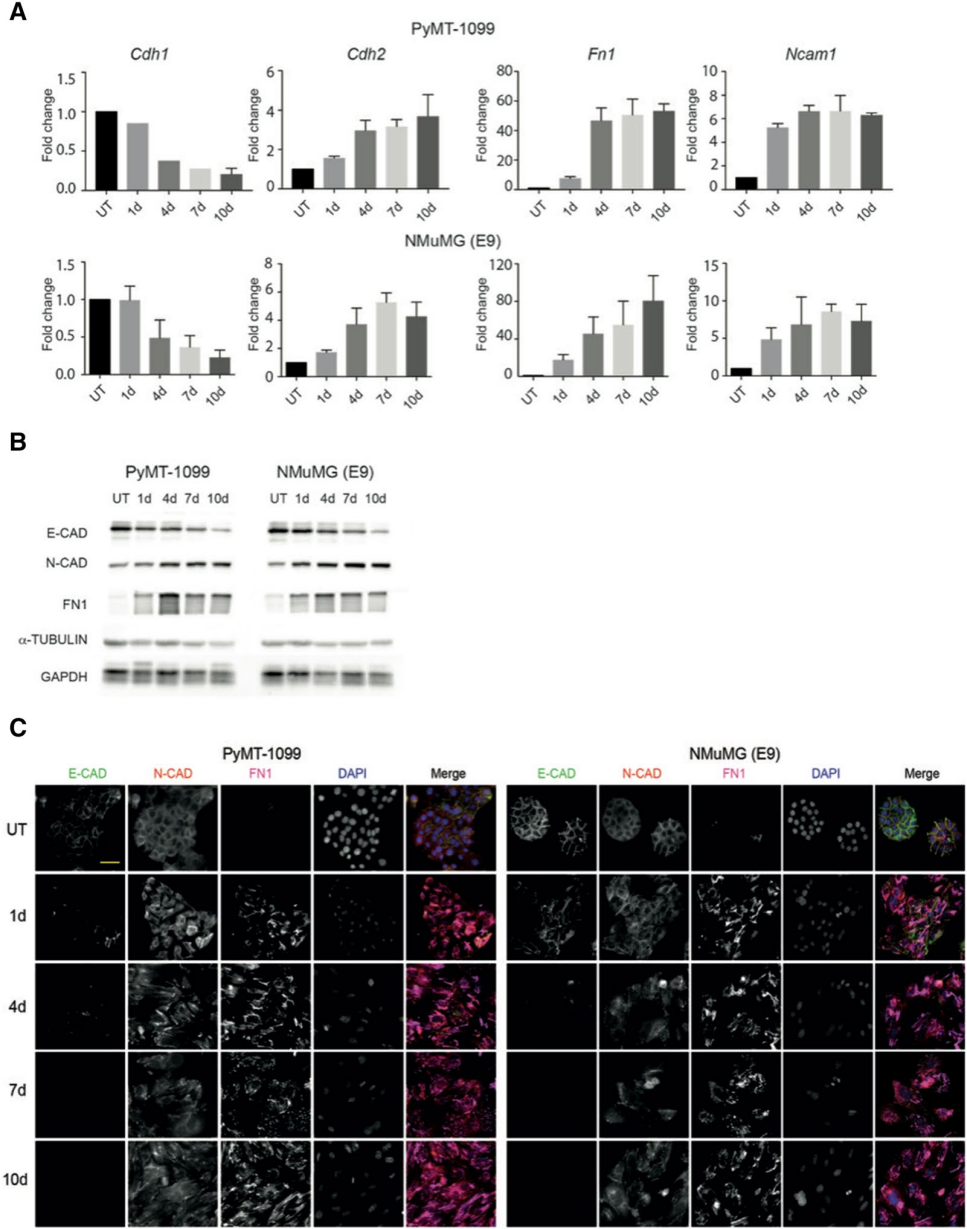
Figure 1. TGFβ-induced EMT in PyMT-1099 and NMuMG cells. PyMT-1099 (top of panel) and NMuMG (E9) (bottom of panel) cells were in parallel treated with TGFβ for 0 (UT), 1, 4, 7 or 10 days.** (A)** RNA isolated from the cells was subjected to quantitative RT-PCR analyses of EMT markers. Graphs represent the relative RNA expression levels of epithelial marker,* Cdh1* and mesenchymal markers* Cdh2*,* Fn1* and* Ncam1* normalized the housekeeping gene* Rpl19*; n = 3.** (B)** Immunoblotting analyses was performed to assess the protein expression levels of epithelial marker E-CAD and mesenchymal markers N-CAD and FN1. α-TUBULIN was used as the loading control; n = 3. All samples were run in parallel on the same gel. Uncropped immunoblot scans from main blots are displayed in Fig. S5.** (C)** Immunofluorescence analysis was performed to assess the expression and/or localization of EMT markers E-CAD, N-CAD and FN1; n = 3. DAPI was used as a nuclear counterstain. Scale bar, 50 μm.

Additionally, the accession number under the ‘Data Availability’ section was incorrectly provided:

“The datasets generated and/or analyzed during the current study are deposited at Gene Expression Omnibus (GEO, accession numbers: GSE112797 (NMuMG (E9) EMT RNA-Seq data); GSE117474 (NMuMG (E9) MET RNA-Seq data); GSE1145722 (PyMT-1099 EMT and MET RNA-Seq data).”

should read:

“The datasets generated and/or analyzed during the current study are deposited at Gene Expression Omnibus (GEO, accession numbers: GSE112797 (NMuMG (E9) EMT RNA-Seq data); GSE117474 (NMuMG (E9) MET RNA-Seq data); GSE114572 (PyMT-1099 EMT and MET RNA-Seq data).”

