# Supplementary material for: PyMT-1099, a versatile murine cell model for EMT in breast cancer
Source: Sci Rep. 2018 Aug 14;8:12123. doi: 10.1038/s41598-018-30640-1 (PMC6092323; doi:10.1038/s41598-018-30640-1)
Supplement: Supplementary file 1 — Supplementary Information [file 41598_2018_30640_MOESM1_ESM.pdf]

Supplementary Information

PyMT-1099, a versatile murine cell model for EMT in breast cancer

Meera Saxena*, Ravi Kiran Reddy Kalathur, Melanie Neutzner, and Gerhard Christofori*

Department of Biomedicine, University of Basel, Mattenstrasse 28, 4058 Basel, Switzerland

*Corresponding authors: Meera Saxena and Gerhard Christofori
Department of Biomedicine
University of Basel
Mattenstrasse 28
4058 Basel
Switzerland
E-mails: gerhard.christofori@unibas.ch
meera.saxena@unibas.ch

Co-authors' e-mail addresses:
Ravi Kiran Reddy Kalathur: ravikiranreddy.kalathur@unibas.ch
Melanie Neutzner: melanie.neutzner@unibas.ch

Supplementary Figures

Figure S1

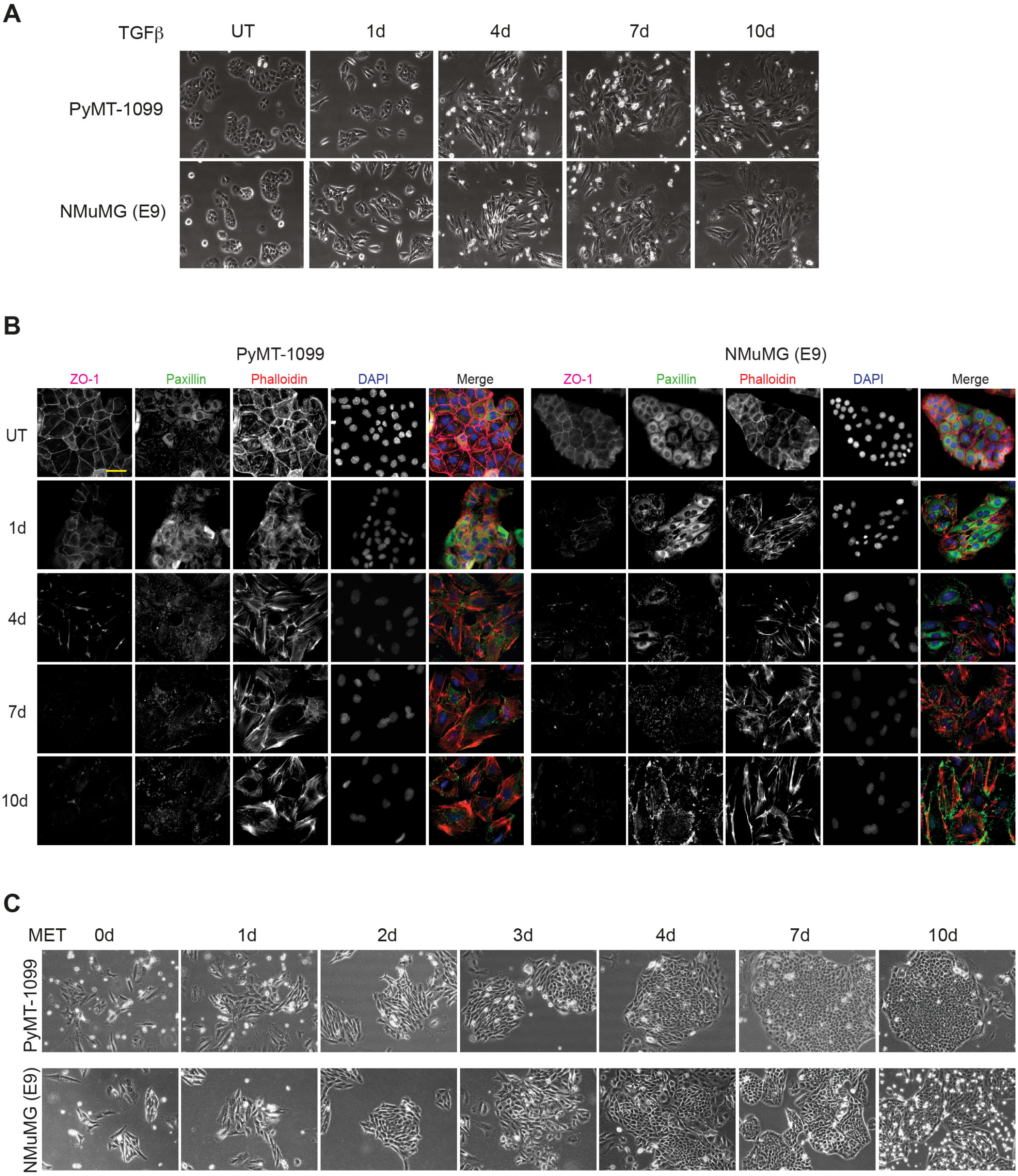

Figure S1 (relates to Figure 2). Time course of a TGFβ-induced EMT and MET in PyMT-1099 and in NMuMG cells.

PyMT-1099 and NMuMG (E9) cells were in parallel treated with TGFβ for 0 (UT), 1, 4, 7 or 10 days. For MET, TGFβ was withdrawn from 10 days treated cells, and monitored for EMT reversal at the indicated time-points.

(A) Photomicrographs represent the morphology of PyMT-1099 and NMuMG (E9) undergoing a TGFβ-induced EMT. Magnification, 10 X.

(B) Immunofluorescence analysis was performed to assess the expression and/or localization of EMT markers ZO-1, focal adhesions (Paxillin) and actin stress fibers (Phalloidin); n=3. DAPI was used as a nuclear counterstain. Scale bar, 50 µm.

(C) Photomicrographs represent the morphology of PyMT-1099 and NMuMG (E9) undergoing MET upon TGFβ withdrawal. Magnification, 10 X.

Figure S2

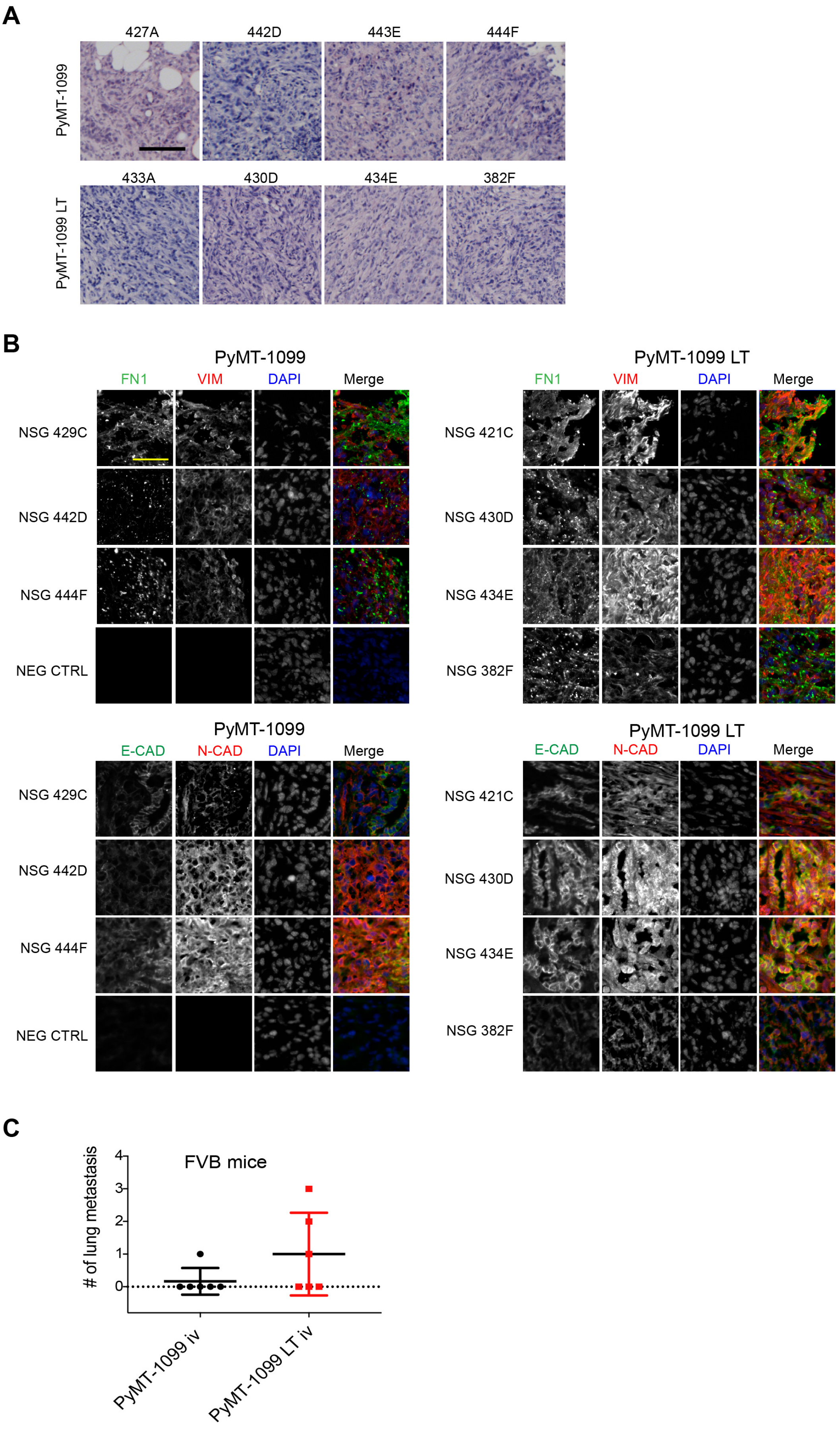
A
427A
442D
443E
444F
PyMT-1099
433A
430D
434E
382F
PyMT-1099 LT
B
PyMT-1099
FN1
VIM
DAPI
Merge
NSG 429C
NSG 442D
NSG 444F
NEG CTRL
PyMT-1099 LT
FN1
VIM
DAPI
Merge
NSG 421C
NSG 430D
NSG 434E
NSG 382F
PyMT-1099
E-CAD
N-CAD
DAPI
Merge
NSG 429C
NSG 442D
NSG 444F
NEG CTRL
PyMT-1099 LT
E-CAD
N-CAD
DAPI
Merge
NSG 421C
NSG 430D
NSG 434E
NSG 382F
C
FVB mice
# of lung metastasis
4
3
2
1
0
PyMT-1099 iv
PyMT-1099 LT iv

Figure S2 (relates to Figure 4). Histology and EMT marker expression in primary tumors of PyMT-1099 and PyMT-1099 LT cells.

(A) Untreated PyMT-1099 and PyMT-1099 LT cells were injected orthotopically into the mammary fat pads of NSG mice. H&E pictures represent the histology of primary tumors resected from NSG mice bearing tumors formed by PyMT-1099 or PyMT-1099 LT cells from the experiment described in Figure 4A. Representative pictures are shown from tumors of 4 out of the 6 mice used in the experiment.

(B) Immunofluorescence analysis was performed to assess the expression of the EMT markers FN1, VIM, E-CAD and N-CAD in tumors formed by PyMT-1099 or PyMT-1099 LT cells in NSG mice from the experiment described in Figure 4A. DAPI was used as a nuclear counterstain. Representative pictures are shown from tumors of 2 out of the 6 mice used in the experiment. Scale bar, 100μm.

(C) The graph represents the number of lung metastases formed in FVB/N mice injected with PyMT-1099 or PyMT-1099 LT cells through the tail vein; n=6. The mice were sacrificed 8 weeks post-injection and the lungs were resected for analysis of cancer cell colonization/ metastatic outgrowth.

Figure S3

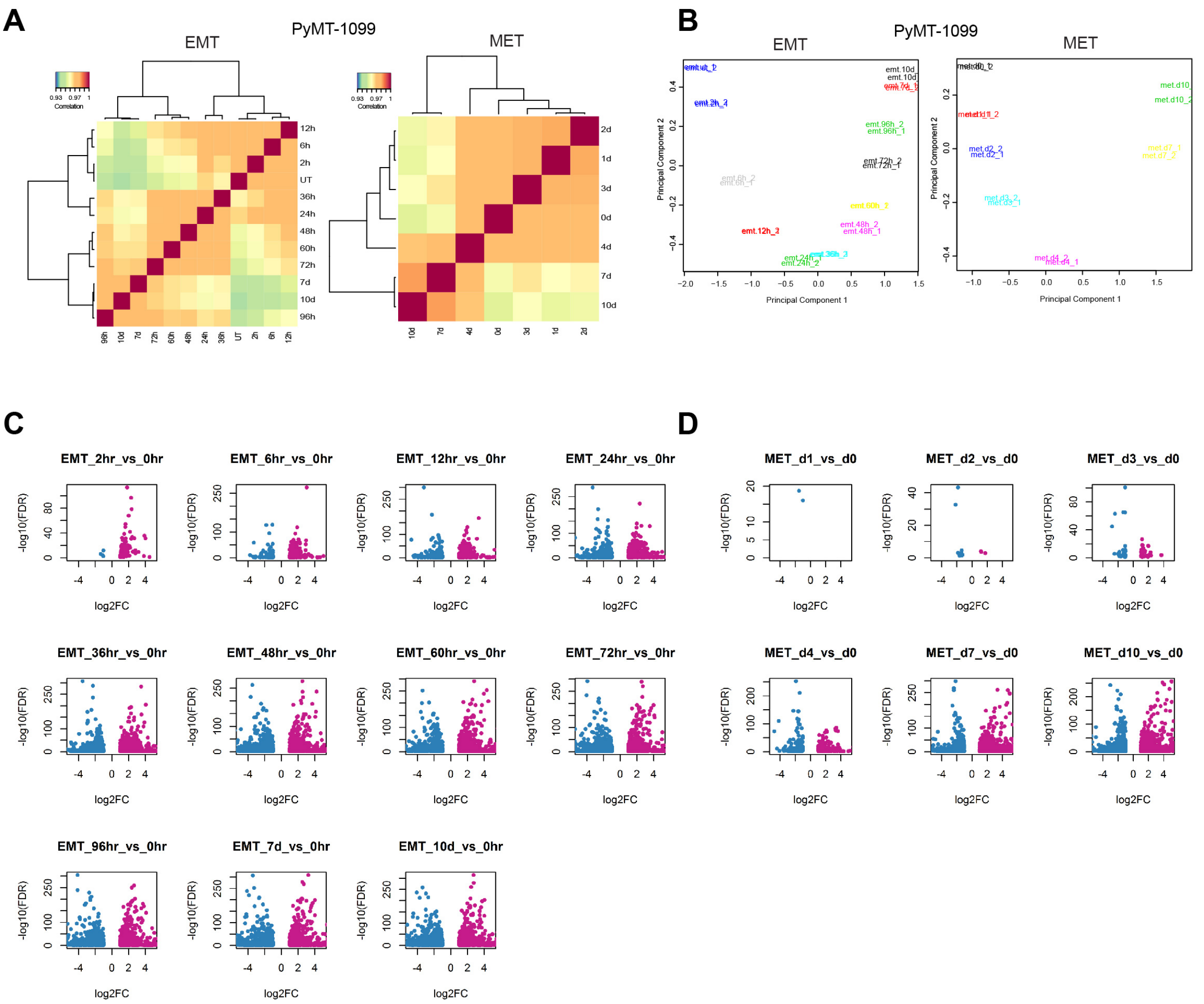

Figure S3 (relates to Figure 5). Gene expression analysis of time courses of EMT and MET in PyMT-1099 cells.

(A) The heatmaps represent the correlation analysis of the biological duplicates of RNA samples from the TGFβ EMT time course and the MET time course in PyMT-1099 cells that were subjected to RNA-Seq.

(B) Shown is the PCA analysis of the RNA samples from the TGFβ EMT time course and the MET time course in PyMT-1099 cells.

(C) Volcano plots depict the differentially regulated genes at each time point of TGFβ treatment of PyMT-1099 cells during EMT.

(D) Volcano plots depict the differentially regulated genes at each time point of PyMT-1099 cells during MET upon TGFβ withdrawal.

Figure S4

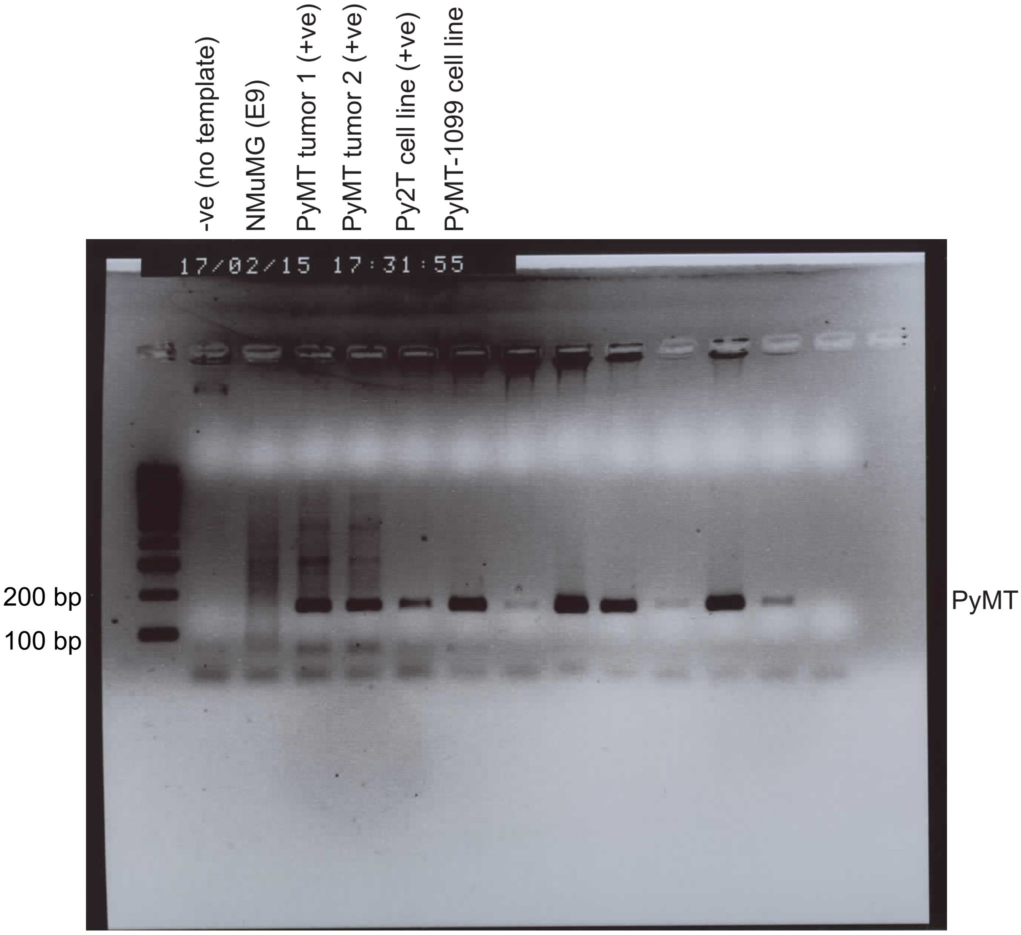

Figure S4. Source data- Uncropped scans from the genotyping PCR (agarose gel) presented in main Fig. 1

Figure S5

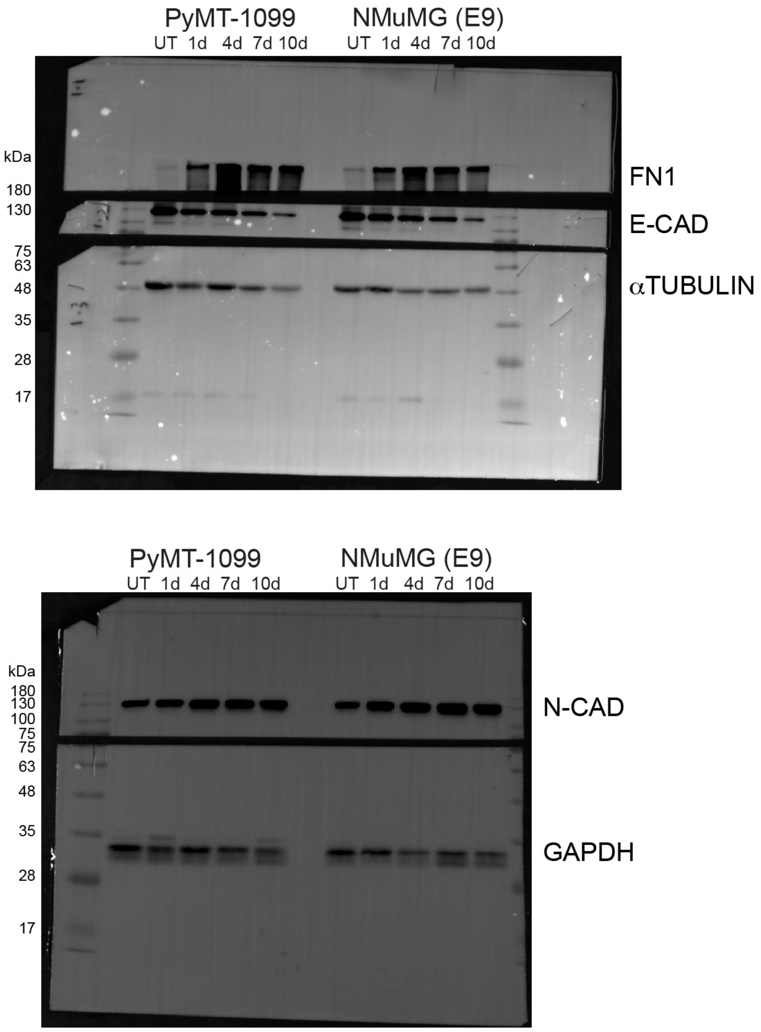

Figure S5. Source data- Uncropped scans from the immunoblotting results presented in main Fig. 2

Table S1

**For genotyping**		
	**Forward (5'-3')**	**Reverse (5'-3')**
PyMT transgene	CGGCGGAGCGAGGAACTGAGG	TCAGAAGACTCGGCAGTCTTAG
**For qPCRs**		
Cdh1	CGACCCTGCCTCTGAATCC	TACACGCTGGGAAACATGAGC
Cdh2	CAATGACGTCCACCCTGTTCT	CTGCCATGACTTTCTACGGAGA
Fn1	CCCAGACTTATGGTGGCAATT	AATTTCCGCCTCGAGTCTGA
Ncam	CAGGAGTCCTTGGAATTCAT	TGGAGAAGACGGTGTGTCTG
